# Supplementary material for: Major trauma presentations and patient outcomes in English hospitals during the COVID-19 pandemic: An observational cohort study
Source: PLoS Med. 2023 Jun 14;20(6):e1004243. doi: 10.1371/journal.pmed.1004243 (PMC10309989; doi:10.1371/journal.pmed.1004243)
Supplement: S1 Appendix — (DOCX) [file pmed.1004243.s001.docx]

S1 Appendix: Study Protocol

A study protocol for a retrospective cohort study and interrupted time series analysis to assess the effect of the COVID-19 pandemic on major trauma presentations and patient outcomes in English hospitals.

Carl Marincowitz1
Omar Bouamra2
Tim Coates3
Dhushy Kumar4
David Lockey5
Lyndon Mason6
Virginia Newcombe7
Julian Thompson8
David Yates2
Fiona Lecky1,2

1. Centre for Urgent and Emergency Care Research (CURE), Health Services Research School of
Health and Related Research, University of Sheffield, Regent Court, 30 Regent Street,
Sheffield, S1 4DA, UK, Fax: +44 (0)114 222 0749 Tel: (+44) (0)114 222 4345, Email:
c.marincowitz@sheffield.ac.uk
2. Trauma Audit Research Network, University of Manchester, Manchester, UK
3. Emergency Medicine Academic Group, Department of Cardiovascular Sciences, University of
Leicester, University Road, Leicester LE1 7RH, UK
4. Department of Critical Care, Anaesthesia and Pre-hospital Emergency Medicine, University
Hospital Coventry, Coventry, UK
5. London Air Ambulance, Royal London Hospital, Whitechapel Road, London, E1 1BB, UK and
North Bristol NHS Trust, Southmead Way, Bristol, BS10 5NB, UK
6. Liverpool University Hospitals NHS Foundation Trust, University of Liverpool, Liverpool, UK
7. Division of Anaesthesia, University of Cambridge, Cambridge, UK
8. Department of Anaesthesia and Intensive Care Medicine, Southmead Hospital Intensive Care
Unit, Southmead Hospital, North Bristol NHS Trust, Bristol, UK

**Abstract**
A protocol for a retrospective cohort study and interrupted time series analysis to investigate the effect of successive COVID related “lockdown” restrictions on major trauma presentations and patient outcomes in English hospitals. The study specifically aims to assess: 1) The impact of successive “lockdowns” on the volume, demographics, injury mechanism, severity, treatment and outcomes of major trauma in England. 2) If the implementation of “lockdowns” affected major trauma related mortality.

A patient cohort will be derived from the Trauma and Audit Research Network (TARN) database, for all trauma receiving hospitals in England, between 1st of January 2017 to 1st of September 2021. This period encompasses two national “lockdown” periods (23rd March 2020 to 29th June 2020 and 2^nd^ Nov 2020 to 16th May 2021) in England. A time series will be used to illustrate changes in the volume and mechanism of injury associated with successive “lockdowns”. Demographic characteristics and features of the clinical care pathways will be compared during the “lockdown” and equivalent pre-COVID periods. To specifically assess if there were any changes in risk adjusted mortality associated with the “lockdowns” interrupted time series analysis will be conducted.

**Background**
To control transmission of COVID-19 during the pandemic the Government of the United Kingdom (UK) implemented successive “lockdown” measures in England.1 The devolved nations (Scotland, Wales and Northern Ireland) introduced similar measures but at different times. In England, the first “lockdown” was announced on the 23rd of March 2020 and, following a period of relaxation, a second “lockdown” was announced on the 30th October due to the emergence of the Alpha variant. This led to a 16% reduction in road traffic, compared to an equivalent pre-pandemic period.2 This has been argued to present a unique opportunity to assess the impact of potential road traffic reducing public health measures on major trauma.3 However, there are also concerns that restrictions associated with “lockdowns” may have contributed to increased non-accidental injury, domestic violence and self-harm related to deteriorating mental health.4-6 Internationally, there is also evidence that despite “lockdown” measures, diversion of health care resources to treating patient with COVID-19, particularly intensive care capacity, may have led to worse outcomes for severely traumatically injured patients.7

Within the context of the UK there have been single centre or regional assessments of the impact of the first “lockdown” on the volume and characteristics of major trauma presentations.8-11 There has been no previous national evaluation of the impact of successive “lockdowns” on major trauma presentations and outcomes in the UK.

**Aims**
To assess the impact of successive “lockdowns” on the volume, demographics, injury mechanism (particularly self-harm, interpersonal violence, other types of non-accidental injury and road traffic accidents) , severity, treatment and outcomes of major trauma in England.
To assess if the implementation of “lockdowns” affected major trauma related mortality.

**Methods**
*Study design*
A retrospective cohort study and interrupted time series analysis using Trauma and Research
Network (TARN) data for England.

*Data set*

Routinely collected data for all TARN submitting hospitals in England for the period 1st of January 2017 to 1st of September 2021 will be used. This period encompasses two “lockdown” periods (23^rd^ March 2020 to 29th June 2020 and 2nd Nov 2020 to 16th May 2021), a prolonged pre-COVID period to establish baseline trend and the period between the two “lockdowns”.
All trauma receiving hospitals (major trauma centres and trauma units) in England submit data on eligible trauma patients to the TARN database for the purposes of audit, governance and
benchmarking. The TARN database includes patients of any age who sustain injury resulting in: hospital admission > 72hrs, critical care admission, transfer to a tertiary/specialist centre or death within 30 days. Isolated femoral neck or single pubic ramus fracture in patients > 65 years and simple isolated injuries are excluded. After study inclusion, a dataset of prospectively recorded variables covering demographics plus injury-related physiological, investigation, treatment and outcome parameters are collated using a standard web-based case record form by TARN hospital audit coordinators. Injury descriptions from imaging, operative and necropsy reports are submitted by TARN coordinators - all injuries are coded centrally using the Abbreviated Injury Scale, this enables calculation of the Injury Severity Score (ISS).

*Inclusion Criteria*
All TARN eligible patients identified between the 1st of January 2017 to 1st of September 2021.

*Analysis*To illustrate changes in the volume and mechanism of injury associated with successive
“lockdowns”, a quarterly time series for the period 1st of January 2017 to 1st of September 2021 will be conducted for the total volume of major trauma in England. The time series will be further stratified by management in a major trauma centre (MTC), road traffic accidents, intentional injuries (further stratified into types of interpersonal violence, paediatric non-accidental injury and self-harm) and other types of unintentional injury.

Demographic characteristics of TARN eligible patients including age, gender, physiology, injury
severity and body region injury will be compared for the first “lockdown” (24th March to 3rd July
2020 inclusive) and second lock down (1st November 2020 to 16th May 2021 inclusive) and will also be compared to equivalent pre-COVID-19 periods in 2018-2019. Similarly, to assess changes in management pathways for patients, the total and proportion of traumatically injured patients who were received by or transferred to a MTC, assessed by a consultant in the Emergency Department, received CT imaging, underwent an operation, were admitted ICU and or died in hospital will be compared in the pre-COVID and “lockdown” time periods.

To specifically assess if there were any changes in risk adjusted mortality associated with “lockdown” a weekly time series of risk adjusted mortality rate per 100, 000 will be plotted for the period 29^th^ October 2018 to 16th May 2021. The weekly W statistics will be calculated for each consecutive weekly period using the conventional TARN method. The W can be interpreted as the number of excess survivors per 100 patients (observed – expected given case mix). Interrupted times series (ITS) analysis will be conducted to assess the impact of the “lockdowns” on the baseline trend of risk adjusted mortality. A segmented regression model predicting the weekly risk adjusted mortality will be estimated and a discontinuity in the gradient (trend) or intercept (level) of the fitted model will be tested for at the weekly time point of implementation of each “lockdown” (24th March 2020 and 2nd November 2020) and at the time of relaxation of the first “lockdown” (29th June). Dates are chosen to incorporate the week each policy was implemented.

**Ethical, Regulatory Considerations and Dissemination**
This analysis will be conducted on fully anonymised Trauma Audit and Research Network (TARN) data. The UK Health Research Authority Patient Information Advisory Group (PIAG) has given approval (Section 251) for analysis of anonymised Trauma Audit and Research Network (TARN) data. Details of the Section 251 approval are available here: https://www.tarn.ac.uk/Content.aspx?ca=2&c=3857.

We intend to present the results of this study at conferences and to publish the results in a peer scientific journal.

**Study Team**
The research team includes a range of clinical and statistical methods experts on the evaluation of trauma care in the UK. FL and TC are professors of Emergency Medicine and DSK is a consultant in critical care who have collaborated on a range of TARN and other trauma related clinical trials and other research projects. They have permanent positions in TARN (FL is Research Director, DSK is director of clinical audit and TC is chairman of the TARN executive board). OB is the TARN permanent medical statistician. DY is a professor emeritus of Emergency medicine and member of the TARN research committee. CM is an NIHR Clinical Lecturer in Emergency Medicine and member of the TARN research committee co-ordinating this project.
External investigators providing additional expertise include: DJL intensive care consultant and Hon. Professor of Trauma & Pre-hospital Emergency Medicine, JT intensive care consultant and Major Trauma Network Research Lead. LM associate professor and consultant in Trauma and Orthopaedics and VN is an Honorary Consultant in Neurosciences and Trauma Critical Care and Emergency Medicine and Royal College of Emergency Medicine Associate Professor.

**Risks and anticipated benefits for trial participants and society**
The study will use existing fully anonymised data derived from clinical routinely collected for the purposes of audit of trauma care and not alter patient management. The risks to patients involved in the study are therefore very low and principally relate to data protection and confidentiality.

Future trauma patients and society in general will benefit from an evaluation of whether changes in behaviour as result of “lockdown” measures affected presentations and outcomes related to major trauma. The results may also inform future NHS decision making about the balance between continuing routine care and responding to public health emergencies. Such information could also inform public health interventions aimed at reducing trauma related to road traffic accidents and non-intentional injury.

**Data availability statement:**
Data may be obtained from a third party and are not publicly available. The de-identified patient
data used for this study are the property of the Trauma Audit and Research Network (TARN), based at the University of Manchester. These data may be requested directly from TARN.

**Competing Interests**
The authors have declared no competing interest.

**Funding**
This study did not receive any direct funding and has been internally commissioned by the Trauma Audit and Research Network (TARN) research committee.

1. https://www.instituteforgovernment.org.uk/sites/default/files/timeline-lockdown-web.pdf.
2. https://www.gov.uk/government/statistics/provisional-road-traffic-estimates-great-britainoctober-2020-to-september-2021/provisional-road-traffic-estimates-great-britain-october-
2020-to-september-2021.
3. Laverty AA, Millett C, Majeed A, Vamos EP. COVID-19 presents opportunities and threats to
transport and health. Journal of the Royal Society of Medicine. 2020;113:251-254.
4. Olding J, Zisman S, Olding C, Fan K. Penetrating trauma during a global pandemic: Changing
patterns in interpersonal violence, self-harm and domestic violence in the Covid-19
outbreak. The Surgeon. 2021;19:e9-e13.
5. Niedzwiedz CL, Green MJ, Benzeval M, et al. Mental health and health behaviours before
and during the initial phase of the COVID-19 lockdown: longitudinal analyses of the UK
Household Longitudinal Study. J Epidemiol Community Health. 2021;75:224-231.
6. Bradbury-Jones C, Isham L. The pandemic paradox: The consequences of COVID-19 on
domestic violence. Journal of clinical nursing. 2020.
7. Driessen MLS, Sturms LM, Bloemers FW, et al. The Detrimental Impact of the COVID-19
Pandemic on Major Trauma Outcomes in the Netherlands: A Comprehensive Nationwide
Study. Annals of Surgery. 2022;275.
8. Hampton M, Clark M, Baxter I, et al. The effects of a UK lockdown on orthopaedic trauma
admissions and surgical cases. Bone & Joint Open. 2020;1:137-143.
9. Rajput K, Sud A, Rees M, Rutka O. Epidemiology of trauma presentations to a major trauma
centre in the North West of England during the COVID-19 level 4 lockdown. European
journal of trauma and emergency surgery. 2021;47:631-636.
10. Dass D, Ramhamadany E, Govilkar S, et al. How a pandemic changes trauma: Epidemiology
and management of trauma admissions in the UK during COVID-19 lockdown. Journal of
Emergencies, Trauma, and Shock. 2021;14:75.
11. Adiamah A, Thompson A, Lewis-Lloyd C, et al. The ICON Trauma Study: the impact of the
COVID-19 lockdown on major trauma workload in the UK. European journal of trauma and
emergency surgery. 2021;47:637-645.
