# Supplementary material for: Major trauma presentations and patient outcomes in English hospitals during the COVID-19 pandemic: An observational cohort study
Source: PLoS Med. 2023 Jun 14;20(6):e1004243. doi: 10.1371/journal.pmed.1004243 (PMC10309989; doi:10.1371/journal.pmed.1004243)
Supplement: S1 Checklist — (DOCX) [file pmed.1004243.s002.docx]

S1 Checklist: Strobe Statement

STROBE Statement—Checklist of items that should be included in reports of ***cohort studies***

|  | Item No | Recommendation |
| --- | --- | --- |
| **Title and abstract** | 1 | (*a*) Indicate the study’s design with a commonly used term in the title or the abstract  Page 1 (Title) |
|  |  | (*b*) Provide in the abstract an informative and balanced summary of what was done and what was found  Page 2 (abstract) |
| Introduction | | |
| Background/rationale | 2 | Explain the scientific background and rationale for the investigation being reported  Page 3 (Introduction paragraphs 1-3) |
| Objectives | 3 | State specific objectives, including any prespecified hypotheses  Page 3 (Introduction paragraph 4) |
| Methods | | |
| Study design | 4 | Present key elements of study design early in the paper  Page 3-4 (Methods paragraph 1) |
| Setting | 5 | Describe the setting, locations, and relevant dates, including periods of recruitment, exposure, follow-up, and data collection  Page 4 (Methods, data set section) |
| Participants | 6 | (*a*) Give the eligibility criteria, and the sources and methods of selection of participants. Describe methods of follow-up  Page 4 (Methods, data set section) |
|  |  | (*b*) For matched studies, give matching criteria and number of exposed and unexposed  *NA* |
| Variables | 7 | Clearly define all outcomes, exposures, predictors, potential confounders, and effect modifiers. Give diagnostic criteria, if applicable  Page 4-5 (Methods, analyses section) |
| Data sources/ measurement | 8* | For each variable of interest, give sources of data and details of methods of assessment (measurement). Describe comparability of assessment methods if there is more than one group  Page 4 (Methods, data set section) |
| Bias | 9 | Describe any efforts to address potential sources of bias  Page 4-5 (Methods, analyses section) |
| Study size | 10 | Explain how the study size was arrived at  Figure 1 |
| Quantitative variables | 11 | Explain how quantitative variables were handled in the analyses. If applicable, describe which groupings were chosen and why  Page 4-5 (Methods, analyses section) |
| Statistical methods | 12 | (*a*) Describe all statistical methods, including those used to control for confounding Page 4-5 (Methods, analyses section) |
|  |  | (*b*) Describe any methods used to examine subgroups and interactions NA |
|  |  | (*c*) Explain how missing data were addressed NA |
|  |  | (*d*) If applicable, explain how loss to follow-up was addressed NA |
|  |  | (*e*) Describe any sensitivity analyses  Page 4-5 (Methods, analyses section) |
| Results | | |
| Participants | 13* | (a) Report numbers of individuals at each stage of study—eg numbers potentially eligible, examined for eligibility, confirmed eligible, included in the study, completing follow-up, and analysed Figure 1 |
|  |  | (b) Give reasons for non-participation at each stage Figure 1 |
|  |  | (c) Consider use of a flow diagram  Figure 1 |
| Descriptive data | 14* | (a) Give characteristics of study participants (eg demographic, clinical, social) and information on exposures and potential confounders Table 1 |
|  |  | (b) Indicate number of participants with missing data for each variable of interest Table 1 |
|  |  | (c) Summarise follow-up time (eg, average and total amount)  Table 1 |
| Outcome data | 15* | Report numbers of outcome events or summary measures over time  Figure 2-6 |
| Main results | 16 | Give unadjusted estimates and, if applicable, confounder-adjusted estimates and their precision (eg, 95% confidence interval). Make clear which confounders were adjusted for and why they were included  Tables 1 and 2 |
|  |  | (*b*) Report category boundaries when continuous variables were categorized Tables 1 and 2 |
|  |  | (*c*) If relevant, consider translating estimates of relative risk into absolute risk for a meaningful time period  NA |
| Other analyses | 17 | Report other analyses done—eg analyses of subgroups and interactions, and sensitivity analyses  Page 8 and 9 Interrupted time series section |
| Discussion | | |
| Key results | 18 | Summarise key results with reference to study objectives  Page 10 Discussion, Summary section |
| Limitations | 19 | Discuss limitations of the study, taking into account sources of potential bias or imprecision. Discuss both direction and magnitude of any potential bias  Page 11 Discussion, strengths and limitations section |
| Interpretation | 20 | Give a cautious overall interpretation of results considering objectives, limitations, multiplicity of analyses, results from similar studies, and other relevant evidence  Page 11, 12 Discussion, Implications section |
| Generalisability | 21 | Discuss the generalisability (external validity) of the study results  Page 11, 12 Discussion, Implications section |
| Other information | | |
| Funding | 22 | Give the source of funding and the role of the funders for the present study and, if applicable, for the original study on which the present article is based  Submitted to journal |

*Give information separately for exposed and unexposed groups.

**Note:** An Explanation and Elaboration article discusses each checklist item and gives methodological background and published examples of transparent reporting. The STROBE checklist is best used in conjunction with this article (freely available on the Web sites of PLoS Medicine at http://www.plosmedicine.org/, Annals of Internal Medicine at http://www.annals.org/, and Epidemiology at http://www.epidem.com/). Information on the STROBE Initiative is available at http://www.strobe-statement.org.
