# Supplementary figures and images for: Major trauma presentations and patient outcomes in English hospitals during the COVID-19 pandemic: An observational cohort study
Source: PLoS Med. 2023 Jun 14;20(6):e1004243. doi: 10.1371/journal.pmed.1004243 (PMC10309989; doi:10.1371/journal.pmed.1004243)

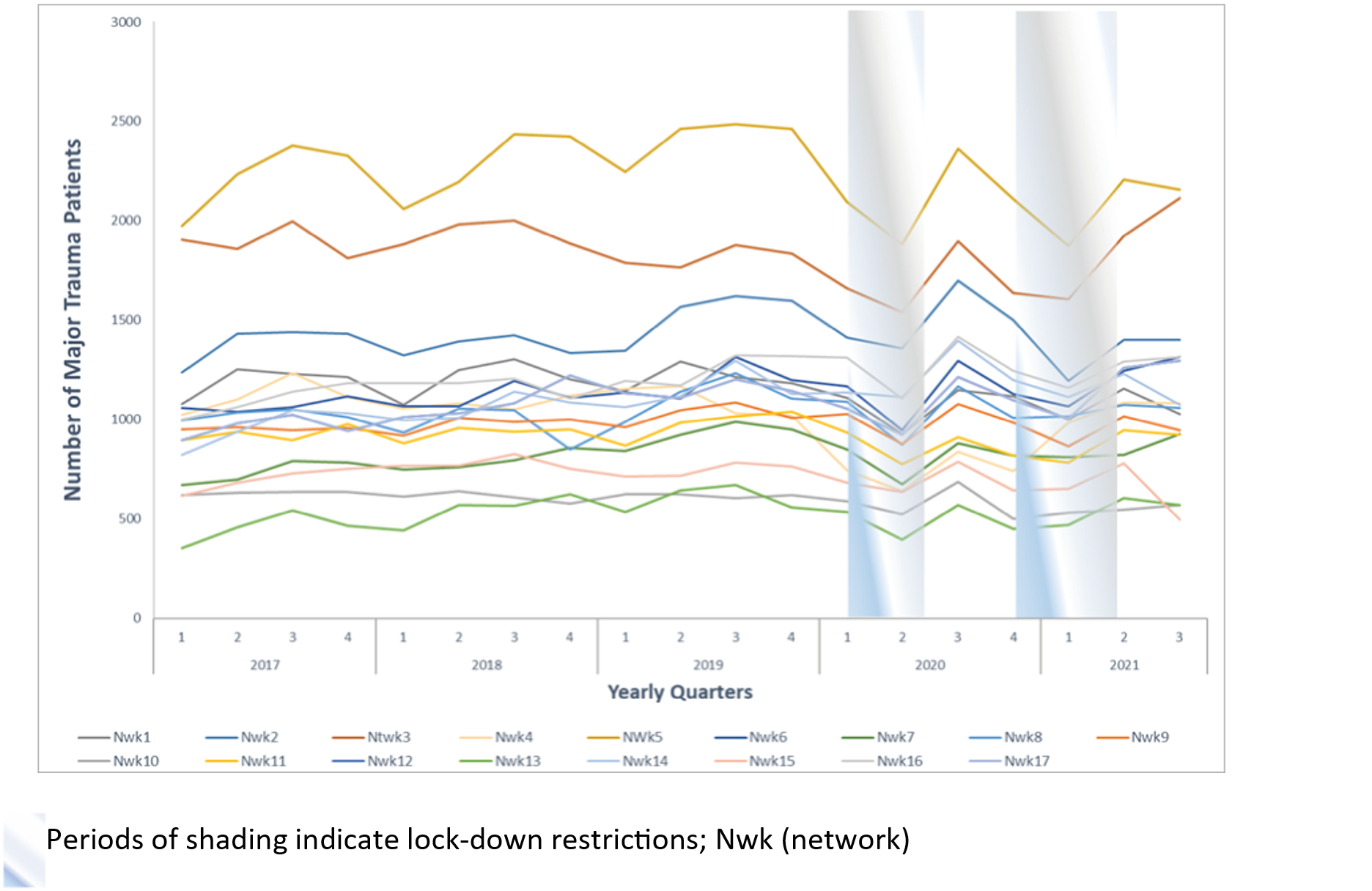

Supplement: S1 Fig — Periods of shading indicate lock-down restrictions. Nwk, network. (DOCX) [file pmed.1004243.s003.docx]
