# Supplementary material for: Major trauma presentations and patient outcomes in English hospitals during the COVID-19 pandemic: An observational cohort study
Source: PLoS Med. 2023 Jun 14;20(6):e1004243. doi: 10.1371/journal.pmed.1004243 (PMC10309989; doi:10.1371/journal.pmed.1004243)
Supplement: Attachment — Submitted filename: Final respone to reviewers comments.docx [file pmed.1004243.s004.docx]

Dear Dr Callam Davidson,

Thank you for the useful and detailed comments and the opportunity to further revise and resubmit our manuscript to PLOS Medicine ("The effect of the COVID-19 pandemic on major trauma presentations and patient outcomes in English hospitals."PMEDICINE-D-22-03493R1).

We hope these revisions and responses meet with your approval and those of the reviewers. We look forward to hearing from you in due course.

Yours Sincerely,

Carl Marincowitz

We have addressed the comments and suggestions, revising our manuscript as detailed below:

-----------------------------------------------------------
Requests from the editors:

*Please revise your title according to PLOS Medicine's style. Your title must be nondeclarative and not a question. It should begin with main concept if possible. "Effect of" should be used only if causality can be inferred, i.e., for an RCT. Please place the study design ("A randomized controlled trial," "A retrospective study," "A modelling study," etc.) in the subtitle (ie, after a colon).*

Response: This has been amended as suggested.

*Please add line numbering to your manuscript to facilitate further review.*

Response: Line numbering has been added.

*Please structure your abstract using the PLOS Medicine headings (Background, Methods and Findings, Conclusions).*

Response: The headings have been amended.

*Abstract Background: Provide the context of why the study is important. The final sentence should clearly state the study question.*

Response: This has been amended.

*Abstract Methods and Findings:

* Please ensure that all numbers presented in the abstract are present and identical to numbers presented in the main manuscript text.*

Response: These have been checked and do correspond.

** In the last sentence of the Abstract Methods and Findings section, please describe the main limitation(s) of the study's methodology.*

Response: This last sentence of abstracts Methods and Findings section now include the main limitations.

*At this stage, we ask that you include a short, non-technical Author Summary of your research to make findings accessible to a wide audience that includes both scientists and non-scientists. The Author Summary should immediately follow the Abstract in your revised manuscript. This text is subject to editorial change and should be distinct from the scientific abstract. Please see our author guidelines for more information:* [*https://journals.plos.org/plosmedicine/s/revising-your-manuscript#loc-author-summary*](https://journals.plos.org/plosmedicine/s/revising-your-manuscript#loc-author-summary)

Response: An Author Summary has been added

*Please remove the ‘What is already know on this subject’ and ‘What this study adds’ sections and replace them with an Author Summary.*

Response: These sections have been removed.

*Please update ‘Background’ to ‘Introduction’.*

Response: This has been amended

*Please place citations in square brackets preceding punctuation throughout.*

Response: This has been amended.

*Please include your prospective protocol with your revised manuscript as a Supporting Information file to be published alongside your study, and cite it in the Methods section. A legend for this file should be included at the end of your manuscript.*

Response: Our protocol is now included in S1 appendix.

*Please add the following statement, or similar, to the Methods:* *"This study is reported as per the Strengthening the Reporting of Observational Studies in Epidemiology (STROBE) guideline (S1 Checklist)."*

Response: This has been included as suggested in the first paragraph of the methods section.

*When completing the checklist, please use section and paragraph numbers, rather than page numbers.*

Response: These have now been added.

*For all observational studies, in the manuscript text, please indicate: (1) the specific hypotheses you intended to test, (2) the analytical methods by which you planned to test them, (3) the analyses you actually performed, and (4) when reported analyses differ from those that were planned, transparent explanations for differences that affect the reliability of the study's results. If a reported analysis was performed based on an interesting but unanticipated pattern in the data, please be clear that the analysis was data-driven*.

Response: We now include the specific hypothesis tested in the final line of the introduction section. We provide the analytical method used to test the hypothesis and clarification of our analysis being conducted in accordance with our protocol in the first paragraph of the methods section.

*Please include [preprint] in reference 12 (see our website for other reference guidelines* [*https://journals.plos.org/plosmedicine/s/submission-guidelines#loc-references*](https://journals.plos.org/plosmedicine/s/submission-guidelines#loc-references)*).*

Response: This has been amended.

*The terms gender and sex are not interchangeable (as discussed in* [*https://www.who.int/health-topics/gender*](https://www.who.int/health-topics/gender)*); please use the appropriate term.*

Response: TARN records biological sex and gender has been changed to sex throughout.

*Please define the abbreviations in your Figures (in the caption if necessary).*

Response: All abbreviations are now defined

*You may use almost any description as the item name of your supporting information as long as it contains an "S" and number. For example, “S1 Appendix” and “S2 Appendix,” “S1 Table” and “S2 Table,” and so forth.  Further information can be found at* [*https://journals.plos.org/plosmedicine/s/supporting-information*](https://journals.plos.org/plosmedicine/s/supporting-information)*.*

Response: This has been amended as requested.

*Please provide titles and legends for all figures (including those in Supporting Information files).*

Response: Titles and legends are provided for all figures, including those in Supplementary Material. All figures, Supplementary material and accompanying legends are listed at the end of the manuscript.

*Please indicate in the figure caption the meaning of the blue shading in Figures 2-5 (and Figure S1). Please also ensure the axes are consistently labelled across all of these figures.*

Response: Captions have been added to indicate periods of shading indicate lockdown restrictions and axes are now labelled consistently.

*Please ensure your Results are consistently reported in the past tense.*

Response: The results have been changed to ensure they are reported in the past tense.

*Please quantify the results using 95% CIs and p values. Tables 1 and 2: Please do not report P < 0.0001; report as P < 0.001.*

Response: Following consultation with the statistician involved in our study, we do not believe it is possible to calculate a confidence interval for the absolute percentage change between the pre-COVID and COVID time periods presented in Tables 1, 2 and in the results, as this percentage can be <0 or >100.

As per reviewer 3’s suggestion (statistical reviewer) we now present the change in the proportion of patients with each characteristic between the pre-COVID and COVID periods with a confidence interval, but no p values related to statistical testing. We have removed the Chi Squared testing on Reviewer 3’s suggestion. The results section has been updated to reflect this.

*Please label the two panels in Figure 4 as A and B and refer to them as such in the main text. Please also adjust the resolution of these images such that they are of comparable quality to the other figures.*

Response: We have amended Figure 4 as requested.

*The lower panel of Figure 4 is unclear, please provide more information in a Figure legend that would allow the reader to interpret this figure without the need to refer to the main text. Please make it clear that the data used to derive this panel are from the Department of Transport and confirm that you have the appropriate permissions to reproduce these here.*

Response: These details are now provided for Figure 4b.

*Please replace the coloured vertical lines in Figure 6 with black dashed lines and make it clear that these lines denote lockdown periods.*

Response: This has been amended with explanation of the dotted lines added to the key.

*Please detail the analysis in the caption of Figure 6.*

Response: These details have been added to the caption.

*In the ‘Interrupted time-series analysis’ section of the Results, the term "trend" is used to refer to a nonsignificant P value. The term trend should be used only when the test for trend has been conducted. Please revise accordingly.*

Response: Use of trend has been revised throughout as suggested.

*In the Discussion, you include ‘as has been suggested in other international studies’ but you only cite one study. Please include further references or update the sentence as needed.*

Response: This sentence has been amended to reflect the single citation.

*Please present and organize the Discussion as follows: a short, clear summary of the article's findings; what the study adds to existing research and where and why the results may differ from previous research; strengths and limitations of the study; implications and next steps for research, clinical practice, and/or public policy; one-paragraph conclusion.*

Response: We have restructured and rewritten the discussion as requested. In particular, the summary of findings section is shorter, we include strengths and limitations and the implications section has been rewritten/restructured as requested.

*Please remove the Contributors, Competing Interest Statement, Funding Statement, and Data availability statement sections from the main text and ensure the relevant information is captured in your answers to the Submission Form Questionnaire.*

Response: These have been removed

*Please use the "Vancouver" style for reference formatting, and see our website for other reference guidelines* [*https://journals.plos.org/plosmedicine/s/submission-guidelines#loc-references*](https://journals.plos.org/plosmedicine/s/submission-guidelines#loc-references)

Response: Vancouver style formatting is used throughout.

Comments from the reviewers:

*Reviewer #1: In this revised manuscript the authors evaluate the influence of the COVID-19 pandemic on the admission rates of major trauma in England. For this they used the TARN database, presenting with major trauma in the years 2017 to 2021. They compared the lock-down periods with the years 2018 and 2019 and evaluated excess survival and mechanisms of injury. They saw a reduction of 21% and 6,7% during the first and second lockdown respectively. This reduction was mainly in road traffic accidents, whereas the number of at home injuries of the elderly increased in the second lock down period. Moreover survival in major trauma was reduced in the first lock-down period.

Methods and statistics used are adequate. The data set is routinely gathered in a standardized well-known process. Also the survival evaluation is done in a standard, earlier published, way.
Tables and figures are adequately designed and provide a good overview of the results, however are complementary to each other.

The discussion is comprehensive and discusses the relevant questions arising form the results, concerning of the available literature.

Concerning the reduction of the likelihood of survival, of course the question arises whether there was a coincidence with a covid infection in these patients, however I presume these data are not available, but could be discussed in my opinion. This was addressed, however, in the limitations.

Limitations are adequately addressed, as well as the implications.*

Response: Thank you, we don’t think any specific changes have been recommended.

*Reviewer #2: Dear Authors,*
*This is a reasonable study. It is unfortunately very sparsely referenced considering how much data is available during the last 2 years on this topic. Even a recent SRMA covered the global impact of the pandemic on major trauma epidemiology (PMID: 35723706).
This UK experience needs to be in context of other regions of the world at least in relation to the first wave, which probably the best documented.*

Response: We now cite the suggested review and meta-analysis to better contextualise our findings.

Reviewer #3: Thanks for the opportunity to review your manuscript. My role is as a statistical reviewer, so my review concentrates on the study design, data, and analysis that are presented. I have put general questions first, followed by queries relevant to a specific section of the manuscript (with a page/paragraph reference).

This study uses a cohort of patients that have experienced serious trauma, with data sourced from a routinely collected health data from trauma centres and units in England, compiled into a trauma registry. The main research question was whether there were changes in hospitalisations for major trauma (with a variety of outcomes examined, including volume, mechanisms, patient type, outcomes) during the first 20 months of COVID. Data was compiled into quarters for the time-series analysis of patient characteristics and care pathways, and an overall test for variation in patient characteristics (chi-square) for across the time periods was used. For changes in risk adjusted survival a weekly time series was used with a segmented regression model, testing for trend and level changes in this outcome. There is a large dataset available - across the study period there were >190 000 patients for the ITS, and >50 000 patients on the analysis of patient characteristics.

*The data and analyses presented mostly match the pre-registered protocol. The two aspects of study design I didn't see in the protocol were the selection of the comparator time periods (to the lockdown periods), and the goodness of fit test for this comparison. Were these specified after the data was available?*

Response: Apologies if not clear, the comparison between lockdown and equivalent pre-lockdown periods is outlined in the second paragraph of the analysis section of the protocol (now included in Supplementary Material 1”: “compared for the first “lockdown” (24th March to 3rd July
2020 inclusive) and second lock down (1st November 2020 to 16th May 2021 inclusive) and will also be compared to equivalent pre-COVID-19 periods in 2018-2019”.

The goodness of fit test for this comparison was included after the protocol was written and data were available. We have now removed this test and the chi squared test and simply report changes in proportion for each characteristic compared with 95% confidence intervals (Tables 2 and 3)

*I think that the application of the chi-square tests to the stratified number of admissions (Table 1) is limited. With even a moderately large sample size the chi-square test is always 'significant', e.g. the % of males in lockdown 1 vs. comparator period 1 is 54.3% vs. 55.4%, and still has p<0.05 with Bonferroni adjustment. I think interpreting the differences here on the absolute change (with CI) is much more informative than using the p-values, and I would consider just presenting the effect estimates with CI.*

Response: As indicated above, following consultation with the statistician involved in our study, we do not believe it is possible to calculate a confidence interval for the absolute percentage change between the pre-COVID and COVID time periods presented in Tables 1, 2 and in the results, as this percentage can be <0 or >100.

We now present the change in the proportion of patients with each characteristic between the pre-COVID and COVID periods in Tables 1 and 2. We have removed the Chi Squared testing. The results section has been updated to reflect this.

*One aspect of the study that I found confusing was the use of two different years for the comparator periods for lockdown 1 and lockdown 2. Was the intention to find the same length of time pre-COVID as each lockdown period to limit seasonal influences on difference between the comparator periods? It does also make it more difficult to interpret whether presence or absence of a change in lockdown 1 vs lockdown 2 is associated with differential effects of the two lockdowns vs. the different comparator periods.*

Response: Lockdown period 1 24^th^ March 2020 – 3^rd^ July 2020 was compared to equivalent pre-lockdown period between 24^th^ March 2019 – 3^rd^ July 2019. Lockdown period 2 1^st^ November 2020 – 16^th^ May 2021 was compared to equivalent pre-lockdown period between 1^st^ November 2018 – 16^th^ May 2019. The second lockdown comparator period of 1^st^ November 2018 – 16^th^ May 2019 was used because if 1^st^ November 2019 – 16^th^ May 2020 had been used as the comparator period then a period of increasing COVID prevalence from March 2020 onwards in England would have been included in deriving the comparator population.

*P4, Paragraph 5. Was the segmented regression model a linear regression model or another distribution? Was there any checks for residual autocorrelation?*

Response: A segmented regression model regression model with the Prais-Winsten adjustment for auto-correlation was used. The details of the segmented regression are provided in 3^rd^ sentence of the last paragraph of the analyses section. We now include use of the Prais-Winsten method in the penultimate sentences of the 3^rd^ paragraph of the analyses section with a reference.

*Figure 2 - could you add a label on the shaded time periods to indicate which lockdown period these are? And put the acronym (MTC) after the phrase in the title?*

Response: We now include a caption indicating periods of shading show lockdown periods and the meaning of MTC.

*Figure 3. I would probably use 'January' if August isn't shortened. Also need a note to explain the 'RTC' acronym.*

Response: The title has been amended and the RTC acronym defined in the figure caption.

*Figure 4. Is there a way to show the traffic index on the same graph as the number of trauma patients (e.g. a secondary axis)?*

Response: We have attempted to present both parts of the figure on one graph and were not able to. We now present this figure as recommended by the journal editors.

*Figure 5. I wasn't sure what 'NAI' acronym was here.*

Response: NAI is now defined in the figure caption.


*Reviewer #4: The authors ought to be congratulated for a well-done analysis across trauma patients in England. Fascinating data and well written paper. Impact of Covid on a mature well defined national trauma system and patient access to optimal care and outcomes. Well done and based on a unique database. Discussion is very well considered. I have no significant issues and enjoyed the paper and the ethical challenges defined. My only remarks concern two statements in the discussion section that I would like the authors to rethink and perhaps elaborate on.*

Response: Thank you.

*In the discussion the authors state that their most important finding for clinical practice is the reduction in adjusted survival in the beginning if the first lockdown. Which was equated to 2 additional deaths per 100 patients. I agree that this most certainly cannot be explained by the reduction in COVID prevalence. They state that this is likely due to reconfiguration and disruption of services, particularly for older patients presenting with low energy mechanisms of injury. Yet, the MS describes that the TARN excludes Isolated femoral neck or single pubic ramus fractures in patients > 65 years and simple isolated injuries.*

Response: Despite the exclusion of Isolated femoral neck or single pubic ramus fractures in patients > 65 years and simple isolated injuries, older adults presenting with low falls are now represent the majority of TARN eligible patients with TBI the commonest cause of death in this group. We now state this in the second paragraph of the implications section.

*It's interesting to see the similarities and differences in trauma occurrence inflicted by imposed covid restrictions between international trauma data sets. I believe the authors have sufficiently compared and evaluated their data with studies performed in other European countries. In my opinion the most interesting statement is that the initial reduction in likelihood of survival could not be attributed to decreased care in major trauma centres or ICU. The authors support this by posing that the proportion of patients admitted to the ICU, by-passed and transferred to major trauma centres, also decreased during the second lockdown period without a corresponding significant decrease in survival. This is plausible, but the authors need to convince us that there is no independent correlation. The study from the Netherlands describes an increased trauma related mortality and risk of death associated with patients with TBI which were not admitted to the ICU. The present study
does not analyze the relationship between TBI and mortality yet describes absolute reductions in moderate and severe traumatic brain injury during the second lockdown. Could this change in trauma patient survival between the first and the second lockdown be attributed to the change in TBI prevalence?*

Response: Thank you for this valid point. We have rephrased our argument and limit it to stating that the initial reduction in likelihood of survival when the first lockdown measures were introduced may not be related to ICU occupancy (penultimate sentence of the second paragraph of the implications section). The interrupted time series analysis does estimate a weekly risk adjusted survival rate which adjusts for severity of injury, patient characteristics and other factors.

Any attachments provided with reviews can be seen via the following link:
