# Supplementary material for: Major trauma presentations and patient outcomes in English hospitals during the COVID-19 pandemic: An observational cohort study
Source: PLoS Med. 2023 Jun 14;20(6):e1004243. doi: 10.1371/journal.pmed.1004243 (PMC10309989; doi:10.1371/journal.pmed.1004243)
Supplement: Attachment — Submitted filename: Final respone to reviewers comments.docx [file pmed.1004243.s005.docx]

Dear Dr Philippa Dodd,

Thank you for the favourable response to our resubmission "Major trauma presentations and patient outcomes in English hospitals during the COVID-19 pandemic: a retrospective cohort study." (PMEDICINE-D-22-03493R2). The remaining editorial and production issues have been addressed as outlined below

Yours Sincerely,

Carl Marincowitz
-----------------------------------------------------------
Requests from Editors:

GENERAL
*Thank you for your detailed and considered responses to previous editor and reviewer comments. Please see below for further comments which we require that you address in full prior to publication.
Throughout, please replace the word ‘retrospective’ with ‘observational’ when describing your study.*

Response: Retrospective has been changed to observational throughout.

TITLE
*Please revise to read as follows, ‘Major trauma presentations and patient outcomes in English hospitals during the COVID-19 pandemic: an observational cohort study.*

Response: The title has been amended as requested.

ABSTRACT
*Line 43 – as above, please replace the word ‘retrospective’ with ‘observational’ when describing your study.*

Response: This has been changed.

*Line 45 - please include additional details of your study population including the total number of patients studied and according to each year of your study. Please also detail some of the demographic characteristics that you refer to.*

Response: The additional details requested have been added.

AUTHOR SUMMARY
*Thank you for including an author summary. The author summary should consist of 2-3 succinct bullet points under each of the headings.
Suggest combining the points at lines 78 and 80 into a single statement.*

Response: These points have been combined.

*Line 87 – suggest ‘appear not to have altered…’ for improved brevity*

Response: This statement has been changed as suggested.

*Line 85 – what do these findings mean? Authors should reflect on the new knowledge generated by the research and the implications for practice, research, policy, or public health. Authors should also consider how the interpretation of the study’s findings may be affected by the study limitations. In the final bullet point of ‘What Do These Findings Mean?’, please describe the main limitations of the study in non-technical language. Please revise in-line with this guidance.*

Response: The final bullet has been revised to provide a non-technical summary of the main limitations.

INTRODUCTION
*The introduction is very brief.  We agree with reviewer #2 (please see below) that more emphasis should be placed on the current existing literature in the introduction such that the reader is not misled regarding the novelty of your study.

Please ensure that you address past research and explain the need for and potential importance of your study. Indicate whether your study is novel and how you determined that. If there has been a systematic review of the evidence related to your study (or you have conducted one), please refer to and reference that review and indicate whether it supports the need for your study.*

Response we have now expanded the third paragraph of the introduction citing and explaining the findings of a recent systematic review on this topic (highlighted by reviewer 2). We explain that our study is the first national evaluation of the impact of lockdowns in England.

METHODS and RESULTS
*Line 120 – as above, please replace the word ‘retrospective’ with ‘observational’ when describing your study.*

Response: Retrospective has been changed throughout.

*Line 170 – please remove the PPI statement from the end of the methods section.*

Response: The PPI section has been removed.

*Line 195 – please ensure accurate use of parentheses it appears as though one maybe be missing and one duplicated.
Line 196 – could the low line be removed here? And the ‘.EPS’*

Response: Thank you for highlighting, the sentences and parentheses have now been corrected. Due to difficulty viewing S3 as an EPS file, it is now provided in a word format.

TABLES
*Table 1 – at times the use of parentheses introduces confusion – the mix of square and circular and the data split across different rows has the potential to cause problems for the reader and detract from your data. For example, age 1, row #3, currently reads, ‘-8 (-6.1%]’ should it not read ‘-8 (-6.1%)’.  Similarly, age <16 row #3 currently reads, ‘[-0.4 (-0.8 to 0]’ should it not read as, ‘[-0.4 (-0.8 to 0)]’
In addition, we also suggest separating upper and lower CI bounds with commas rather than the word ‘to’ or hyphens (as these can be confused with the reporting of negative values) which will help to conserve space i.e. ‘[-0.4 (-0.8, 0)]’.
We note similar issues in table 2 also.
Please check carefully throughout, including the supporting files and revise accordingly.*

Response: Thank you for highlighting this. The tables have now been checked, corrected and amended as suggested.

FIGURES
*Figure 2 – please add ‘(MTC)’ to the text below the figure so depict the abbreviation that you define.*

Response: Apologies, this has been corrected.

*Figure 4b – you refer to areas of shading but I cannot see any areas of shading in my version of the manuscript. Presumably this pertains to figure 4a? Please clarify/revise.*

This figure has been corrected so that the legend referring to periods shading is below figure 4a.

*Figure 6 – what do the solid lines represent here? It appears that the dashed lines represent changes in lockdown restrictions (as per the legend). Should the graphic be adjusted, perhaps positioned horizontally?*

Response: The solid lines are the fitted segmented regression. This is now indicated in the text accompanying Figure 6. The dashed line is now positioned horizontally.

*I was unable to access S3 Figure*

This is now provided as a word document to make it easier to access.

*** Please revise to ensure that all detail from the figures is clearly defined for the reader in an appropriate caption affiliated to the figure such that the reader does not need to refer to the test to interpret the figures ***

REFERENCES
*For in-text reference callouts please ensure that a space precedes the opening parenthesis. For example, line 113, ‘presentations[8-11].’ Should read as follows, ‘presentations [8-11].’ Please check and amend throughout where necessary.
Please also remove spaces between citations, for example line 319, ‘analysis[11, 17-19]’ should read,  ‘analysis [11,17-19]’. Please check and amend throughout where relevant.
In the bibliography – please provide access dates for all web references. Please ensure that reference formatting follows our guidelines which can be found here* [*https://journals.plos.org/plosmedicine/s/submission-guidelines#loc-references*](https://journals.plos.org/plosmedicine/s/submission-guidelines#loc-references) *Journal name abbreviations should be those found in the National Center for Biotechnology Information (NCBI) databases.*

Response: We have checked and amended references throughout as requested.

*SOCIAL MEDIA
To help us extend the reach of your research, please detail any Twitter handles you wish to be included when we tweet this paper (including your own, your coauthors’, your institution, funder, or lab) in the manuscript submission form when you re-submit the manuscript.*

Response: Twitter handles: @CarlMarincowitz @CURE_ScHARR @TARNaudit @vfjn2 @DhushyKumar @TJCoats @Liverpool_Ortho @drlyndonmason @davidlbath

Comments from Reviewers:

Reviewer #2: Dear Authors,
*This is an improved manuscript in terms of discussion. But the existing literature is still just brought up in discussion and not in introduction, which makes the reader think that this is very unique work. The introduction should document on quality existing original and review work and identify the uniqueness of this paper in that context. Some of the existing work would dwarf this labour and the authors should be upfront with it if that the case.*

Response: We have now expanded the third paragraph of the introduction to highlight the findings of previous studies.

*Reviewer #3: Thanks for the revised manuscript and responses to my original queries. This versions addresses all the comments I had form my initial review, I don't have any further questions.*

Response: Thank you.

*Reviewer #4: All reported remarks have been amended appropriately.
I have no further remarks.
Great article.*

Response: Thank you.
